# The Nurse in the University: A History of University Education for South African Nurses: A Case Study of the University of the Witwatersrand

**DOI:** 10.1155/2011/813270

**Published:** 2011-09-07

**Authors:** Simonne Horwitz

**Affiliations:** Department of History, University of Saskatchewan, 9 Campus Drive, Saskatoon, SK, Canada S7N 1H9

## Abstract

This paper charts the history and debates surrounding the introduction of academic, university-based training of nurses in South Africa. This was a process that was drawn out over five decades, beginning in the late 1930s. For nurses, university training was an important part of a process of professionalization; however, for other members of the medical community, nursing was seen as being linked to women's service work. Using the case-study of the University of the Witwatersrand, one of South Africa's premier universities and the place in the country to offer a university-based nursing program, we argue that an historical understanding of the ways in which nursing education was integrated into the university system tells us a great deal about the professionalization of nursing. This paper also recognises, for the first time, the pioneers of this important process.

It is with great pride that Wits can claim to be the first university in South Africa to have understood not only the importance of developing nursing as a profession, but also as a potentially valuable academic discipline informed by research and scholarship when it introduced its nursing diploma in 1937. Since 1937, Nursing Education at Wits has grown from strength to strength…

Professor Loyiso Nongxa, Vice-Chancellor and Principal of the University of the Witwatersrand, Johannesburg, on the occasion of the Department of Nursing's 70th anniversary.

It is perhaps unsurprising that on the 70th anniversary of the founding of the Department of Nursing at the University of the Witwatersrand (Wits) the Vice-Chancellor and Principal of that institution would speak about the department in such glowing terms. It was, indeed, a department which has had a praiseworthy history. It is a history which highlights many of the issues that shaped the incorporation of nursing education into the university system in South Africa. Yet, this it is also a little known history.

The general history of nursing in South Africa has been covered in a fair amount of detail by South African nursing's formidable “first lady” Searle in her 1965 book *The History of the Development of Nursing in South Africa, 1652–1960 * [1]. The book, based on her doctoral degree in sociology, pays a great deal of attention to detail but lacks a strong critical analysis that might help us to understand the competing tensions that shaped the nature of nursing education in South Africa over the 20th and into the 21st Centuries. A more nuanced and contextualised history of nursing in South Africa was published in 1994 by the doyen of South African medical history, Marks [2]. While hers is an excellent work which provides fascinating insight into the ways that the nursing profession has changed over time and how issues of race, class, and gender shaped those changes, it is a very general study. More recently a number of increasingly focused studies have begun to explore specific groups of nurses and how these groups have related to their place within the profession [3, 4]. Finally, a growing number of hospital histories have included chapters on nurses. While these chapters do give a cursory nod towards nursing training, they are focused on the relationship between the nurses and the hospital that is under study [4, 5]. Far less attention has been paid to the academic institutions that were key to the process of training nurses. This might be in part due to a lack of easily available sources. However, this important history can be reconstructed from the available secondary sources and primary material we were able to gather from the Wits Department of Nursing itself. This primary material consisted of departmental documents, talks, and speeches given by members of the department and newspaper clippings they kept. We were also fortunate to have personal communications with a number of the role players in the changing nature of nursing education at Wits. When used critically, these primary sources provide a unique insight into not only the events themselves but also the ways in which the participants saw them and the things that they prioritised.

This paper argues that institutions that are geared to professional training were instrumental in shaping the professional identity of groups such as nurses. Therefore, by focusing on the development of university-based nursing education within a specific setting, we are able to discern important local issues, institutional influences, and the role that individuals play in shaping the history of nursing. These are factors that are fundamentally important to this history but which cannot be given sufficient treatment in broader studies.

Wits in Johannesburg, South Africa, began its life as the Transvaal Technical Institute in 1904 and gained full university status in 1922 when the Medical School came into being [6]. As Vice-Chancellor Nongxa suggested in the quote that opened this paper, Wits was the first of South Africa's universities to launch a diploma in nursing a decade and a half later. This marked an important shift in nursing education in South Africa. 

Formal nursing education has had a long history in South Africa. The first formal training course was set up by an Anglican Sister, Henrietta Stockdale, on the Kimberley diamond mines in 1877. For much of the 19th and early 20th centuries, religious institutions were predominantly responsible for nursing education. While some of the religious orders continued their role in training nurses into the 20th century, training was increasingly being carried out by a patchwork of institutions differing in size and standards. Some of these were private, for-profit concerns maintained by industry, some were partly subsidised and others were totally state run although in no coordinated pattern [1]. It was not until the late 1930s that nursing education entered the universities, and it would be another decade before training was done with any measure of uniformity. 

Universities were reluctant to see nursing added to their choices of professional programmes. Although not overtly stated, it seemed that one of the reasons for this was that nursing in South Africa, like in much of the rest of the world, was considered as an extension of women's domestic labour. This was something that was acceptable in the hospital setting where education was tied to service but not something that was to be offered in the academic setting of the university—still very much a male domain in the interwar years. Searle wrote that

[a]t first the universities considered that nursing was not a fit subject for university study, but in time, as faculties of commerce and departments of social and domestic science were added, the nurses were quick to point out that the attitude of the universities was inconsistent and that nursing was at least as important to the community as “book-keeping” and domestic science, and that it was in fact part of medical science [1].

The changing nature of university course offerings was just one of a combination of local internal and external pressures that led to the incorporation of nursing education at South Africa's universities. One of the most important external pressures leading to the expansion of nursing education was the lack of education and training opportunities in South Africa—especially to train sister tutors. This shortage of nurse educators in turn limited the number of young women who could enter training. During the first few decades of the 20th century, sister tutor posts had to be filled with foreign nurses, predominantly from UK or the few South African women who could afford to travel abroad to receive training [1]. In some cases, sisters or matrons were prevailed upon to take up the role of sister tutors in addition to their other duties, but this was hardly ideal [1]. Many nurse leaders, including Bella Gordon Alexander, who served as the General Secretary of the South African Trained Nurses' Association (SATNA) and Matron of the Johannesburg Hospital during the late 1920s and into the 1930s, argued that the local training of sister tutors was the only way that nurses' training could be extended both in terms of quantity and quality. Alexander was a forthright spokesperson for the recognition of nurses as professionals within the health care system [2]. An important part of this recognition would have been the housing of nursing education within the university setting.

In this battle, nurses needed external support. Early champions of nursing education within South Africa included two of Wits' most renowned professors, Raymond Dart and Phillip Tobias—both of whom taught in the Wits Medical School. The Medical School was founded in 1922. Three years later when Dart, the rather eccentric Australian Professor of anatomy, arrived in Johannesburg, the medical school was still in its infancy. Dart, who was the Dean of Medicine between 1925 and 1943, aimed to develop a strong, comprehensive Faculty of Medicine at Wits. He saw the establishment of an academic nursing programme within the faculty as an important aspect of this [6]. It is, therefore, not surprising that he was open to the calls of SATNA for the establishment of local courses for sister tutors. In 1935, through the joint efforts of Dart, with the South African Medical Council and SATNA, it was agreed that courses for white nurses intending to qualify as sister tutors would be taught at Wits and at the University of Cape Town (UCT). In both cases, the medium of instruction was English. Students were admitted into these courses in 1937 [1, 6]. At Wits, the diploma was first located in the Department of Preventive and Promotive Medicine [7]. It was initially a two-year, part -time, and post-basic course, but it was soon converted into the one year fulltime Diploma in Nursing Education (DNEd) [8]. The first six graduates received their diplomas in March of 1939.

This marked an important recognition of nursing as a profession. Over the years that followed, many pioneer nurses attended this course, including Charlotte Searle and Paddy Harrison, who went on to run the Sister Tutors Diploma and then the B.Sc. at the UCT [7]. Yet, the diploma course remained undersubscribed. Although the diploma course had brought nursing into the university, many white women still felt that nursing was inferior to teaching [9]. Although the course was open to both white and black students, applicants required a matriculation certificate and two years of post-basic experience to be admitted, and few black students had the entry qualifications. The lack of qualifications was a major stumbling block, because many black women saw nursing as a high-status career. Indeed, nursing and teaching were the only professional jobs available to black women until the birth of a democratic South Africa in 1994. Most of the black women entering nursing, at least in the early years, were trained in a mission setting. Later, black nurses trained at segregated nurses' training schools which had various relationships with the universities over their histories. We have written elsewhere, in some detail, about the training and experiences of black nurses. In that body of work we use Baragwanath Hospital, which was a Wits teaching hospital, as case study in order to make a number of broader conclusions about black nursing in South Africa [10, 11].

The 1940s saw both a major shortage of nurses [1] and a growth of the courses being offered at Wits. By the late 1940s, diploma courses (for white nurses only) were added in midwifery and in social, mental, and administrative nursing [12]. The establishment of these courses did not, however, settle concerns about the shortage of nurses in the country. In the mid-1940s, Henry Gluckman, then Minister of Health, appointed an ad hoc committee with the mandate to explore ways of reorganising nursing education in order to increase the number of nurses [13]. Among those on the committee were representatives from the nursing community, representatives of the Ministry, and representatives of universities such as Wits. One of the key discussions revolved around where basic nursing education should be located. While Wits' representatives argued that the way forward was for basic training to be undertaken by the universities, this was not universally accepted, as many nurses felt that it would be difficult and unnecessary to train all nurses at a university level [13]. This issue was not solved until the 1980s. What is interesting about this debate, however, is that it was elements within the nursing establishment who were suggesting that universal university education was not necessary. This argument was emanating from a professional group who had fought to place nursing within the university system as part of a process of professionalization. Although the battle for professionalization was not yet over, it had certainly moved forward, and there was another more pressing issue facing nurses and nursing education in the post-war years: a significant nursing shortage.

The 1950s were characterised by the ongoing problem of the shortage of nurses on the one hand and the establishment of an increasing number of nurses' training programmes within South African universities on the other. Four new diploma courses were added in the following decade, but they were all suspended in 1957 because of a lack of suitable candidates. Between 1957 and 1965, no nurse tutors graduated [7, 12]. This might have seemed like a contradictory process, especially when some of the shortages were blamed on the high entry standards and rigorous examination process demanded by the South African Nursing Council (SANC). Yet, as Shula Marks has argued, it was no coincidence that basic nursing training was incorporated into universities during the 1950s. She suggested that the 1950s was a decade during which nursing leaders deliberately sought to increase the status and qualifications of white nurses. This, Marks argues, was a deliberate response to the increasing number of black nurses who were, from the 1950s, moving into positions of power, especially at hospitals which catered for black patients. As in much of the rest of the world, South Africa attempted to increase the status of nurses by moving courses to university settings and lengthening the duration of training with the thought that this would attract candidates from better backgrounds [2].

While nursing education was expanding and reorganisation was being discussed, another important issue was being debated, one which would fundamentally change the shape of nursing in South Africa. Nursing had been under the control of the South African Medical and Dental Council (SAMDC) since the first trained nurses and midwives were registered in 1891 [2]. However, there was almost constant conflict over the control of nursing. One of the biggest issues was that nurses felt they did not get sufficient representation on the SAMDC. For many, it was imperative that nursing be governed by a separate statutory professional council that would be equal in status to the SAMDC [2].

This was, however, a controversial idea. Many outside of nursing circles criticised the nurses' call for a separate governing body. At Wits, Dart was strongly opposed to the development of separate professional bodies and called for a single South African Medical, Dental, and Nursing Council [14]. Reflecting on the establishment of the SANC from the vantage point of the 1980s, Tobias writes: “Maybe it was a mistake, back in the forties, for the nursing profession to have separated itself from the medical (profession) by the setting up of a SA Nursing Council separate from the SA Medical and Dental Council. On the other hand, that might have been a necessary step in the struggle of the nursing profession to achieve recognition” [15]. 

This was another aspect of nurses' struggle for recognition and professional status. In 1944, SANC came into being through Act no. 45 of 1944. From this point, until the 1980s, the SANC was responsible for nursing education standards and was the primary examining body for nursing examinations [16]. Wits was involved in the newly formed SANC on a number of levels. In addition to the University having a nursing representative, Dart served as a member of the Council from its inception until 1951 [17]. During this time, he was joined at Wits by a young member of staff who would become a leading academic and a champion of academic nursing. Phillip Vallentine Tobias began his career at Wits in 1945 when he was appointed Demonstrator in Histology and Instructor in Anatomy. Tobias' biggest impact on nursing at Wits was his campaigning to get the Department of Nursing Education recognised within the Faculty of Medicine. In Tobias' own words:

During the 1950s and 1960s I noticed, at every meeting of the Board of the Faculty of Medicine, that matters affecting Nursing, Physiotherapy and Occupational Therapy were relegated to the tail-end of the agenda and seldom received as much as five minutes of attention among all three [14].

It was only in the late 1960s that Tobias, who was by this time Professor and Head of the Department of Anatomy, put forward a concrete proposal for dealing with this situation. He proposed that there should be an Assistant Dean to oversee Nursing, Physiotherapy and Occupational Therapy. He hoped that this would create something close to a Sub-Faculty for the Allied Medical Disciplines [14]. Only in 1970 did Professor Francois Daubenton, who was then Dean of the Faculty of Medicine, set up an Allied Medical Disciplines Committee with Tobias as its first chairman [14]. 

For the nursing administration in this period of High Apartheid, the training of white, Afrikaans-speaking nurses took priority. They were the first group to be offered basic nursing training at a university level; initially this was a B.A. in Nursing which was offered at the University of Pretoria beginning in 1955 [16]. The University of Stellenbosch and the University of the Orange Free State, also offering Afrikaans medium courses, were not far behind [2]. The first of the English medium universities to begin basic nursing training within a university setting was the University of Cape Town [12]. Shortly thereafter in 1962, Natal also began to offer an English medium course [18]. Wits lagged behind and only began its basic nursing training programme in 1969. Significantly, when Wits introduced its new programme, it was a B.Sc. (Nursing), the first of its kind in South Africa. This was the forerunner of the degree that is today the Bachelor of Nursing [14].

Even at the time that the degree was being introduced there were differing views on its value within the Wits community. The Professor of Nursing Education, Shirley Williamson, also recalled some opposition to the degree emanating from the Dean of Medicine during the early years [19]. This was in opposition to Tobias' view which was that those within the medical school hierarchy supported the integration [20]. It was difficult to untangle this but what was clear was that the introduction of the degree was debated. Tobias claims: “This was a struggle in academic circles at Senate level to accept this. Course work was made as academic as possible but some nurses felt it would be too difficult” [20]. It is difficult to gauge whether the latter was Tobias' own view or he was reflecting the feeling of nurses outside of the Wits system. In general, it seemed as if the Wits nurses were more positive. For them, it was precisely because of the difficult nature of the courses, and that they were university-based, that they supported them, because it was these characteristics that meant the university-based, basic degree fitted into international norms of professionalization. These nursing leaders were also in support of the academic training of nurses as they found themselves working in increasingly highly specialised modern units for intensive care, tissues transplants, neurology, and plastic surgery [21]. In an interview with the *Rand Daily Mail* in October of 1970, Patricia Schwartz explained: “I feel there is a need for the nurse to move into the university and medical school. She is a practitioner in her own right and needs a wider background. The BSc course involves the natural, biological and human sciences, all of which are intimately linked to nursing” [22]. Nursing candidates themselves also seemed to see the value in the new degree. One commented: “Nursing, with a degree is a better career for a woman than medicine, so I chose this rather than studying to be a doctor” [23]. Another explained: “My parents wanted me to go to university. I wanted to nurse. This combines the two” [23].

This kind of degree would produce highly skilled nurses who might be able to relieve some of the pressure on doctors, yet it also further restricted access to nursing training and would do little to alleviate the nursing shortage [2]. By 1969, only 11.3 per cent of all white student nurses had received their basic nursing education at a university [2].

Although these courses were focused on the education of white nurses, the 1950s through 1970s saw a growing move of black nurses into positions of power, especially at black hospitals where apartheid's policies of separate-but-equal had the effect of opening up new opportunities. we have written elsewhere about the education and challenges of black nurses in South Africa [4, 24]. What is important for the purposes of this paper is that by the mid-1950s, access of black nurses to a university education was becoming increasingly difficult. Apart from the stiff entry requirements which were difficult to meet within the context of Bantu Education, black students were also burdened by the University Education Act (no. 45 of 1959) which required them to get ministerial permission before gaining admission, a process that was both difficult and tedious [25]. Faculty, students, and administrators at Wits strongly opposed this legislation but to no avail [26]. It was only in November of 1985 that Mr Andrew Chiloane became the first black student to successfully complete the B.Sc. Nursing course at Wits [27].

In 1969, a subdepartment of Nursing Education was eventually founded. The new sub-department was housed within the Department of Preventive and Promotive Medicine but had a specific representative on the Medical Faculty Board [7, 28]. This meant that the department was starting to gain a greater say in governing its own future and in the events at the medical school more generally.

The first full-time senior lecturer in the new Wits B.Sc. was Lydia Hebestreit [23]. One of her primary tasks was to develop and shape the new degree [7]. However, Hebestreit was in the position for hardly a year when she resigned to take up the post of Director of Nursing at Baragwanath Hospital. Although Hebestreit was returning to Baragwanath Nursing College where she had served as a lecturer and then principal from 1958 to 1966, it is likely that other factors contributed to her departure. Her successor, Shirley Williamson, speculates that Hebestreit was also frustrated at Wits, because she felt that she was not getting the support she needed to get the nursing department started [29]. In 1975, Hebestreit immigrated to Australia.

Shirley Williamson, who had been principal at the Germiston Nursing College, filled the post vacated by Hebestreit [29]. The second member of this early team was Professor Cora Erasmus. Erasmus was the Germiston Medical Officer of Health and part-time Professor of Preventative and Promotive Medicine at Wits. The third of the triumvirate was Merle de Haan. There have been suggestions that the relationship between Williamson and De Haan was often strained and that they had very different personalities, teaching philosophies, and political outlooks [30]. Williamson, like Searle, seemed to have fitted into the more traditional and conservative mode rooted in the universalist model of Nightingale nursing. De Haan, on the other hand, seemed to have been part of a group of personnel in the medical school, social sciences, and humanities who looked more to a model of social health care and community-orientated primary health care developed by S. L. Kark and E. Kark at Wits in the 1940s [31]. 

While these differences might have made for a slightly uncomfortable working environment, the program seemed to attract candidates right from the beginning. In 1969, there were 30 applicants [32], and 12 women were finally enrolled for the first year of the B.Sc. (Nursing) degree at Wits. It was a four-year degree [32]. Of the twelve entrants, three women graduated in 1972. They were Jill Carruthers, Lorayne Anne van Eyssen, and Janet Alice Wilson [7]. Carruthers went on to be awarded the Queen Vic's gold medal as the most outstanding student of the year in addition to attaining a first-class pass in her midwifery examinations [33]. Lorayne van Eyssen received a second-class pass in the examinations. She worked in district nursing services at Johannesburg General Hospital [33]. Graduates of the new degree would be able to take up posts as staff nurses or ward sisters or to work in special units and stimulate research [21, 23]. If graduates completed an additional post-basic Diploma in Nursing Education, they could then serve as tutors.

While the new program was finding its feet academically, the medical school was finding a place to physically locate it. The very first lectures of the new degree took place at the BG Alexander Training College in Hillbrow. From February 1969, when the university reopened, the new subdepartment moved to the Medical School, which was situated adjacent to the Johannesburg General Hospital, also in Hillbrow. This location was important, as the practical teaching was undertaken by a clinical sister in Johannesburg Hospital until 1973 when the university appointed a clinical tutor. At this point, nursing was housed in the Department of Social and Preventive Medicine. 

Professor Williamson remembered the difficulties of those early days when nursing lecturers had to share not only an office but also desks with staff from the Department of Social and Preventative Medicine [29]. In 1975, Wits' nursing department moved into a prefabricated building on top of the library store in the New Medical School on Esselen Street—above the old Johannesburg General Hospital. By the early 1980s, the physical conditions were much improved as the Department of Nursing moved into its present home in the Medical School in Parktown [7].

As far as the curriculum went, the aim of the B.Sc. was to recognise that nursing embraced the whole sphere of medicine. As such, the B.Sc. followed the models of Physiotherapy and Occupational Therapy, which were already well entrenched at Wits [7]. In their first year of nursing, students studied chemistry, anatomy and microanatomy, psychology or sociology, preventative and promotive health, and nursing [23]. Later years included physiology, more advanced sociology or psychology, microbiology, pathology, and pharmacology, and an increasing amount of time was spent in the hospital wards [23]. During these early years of basic training, Tobias taught the first introductory course on human genetics to be offered to student nurses in South Africa. He also taught elements of anatomy, embryology and histology to student nurses at Wits [14]. 

Along with this varied and demanding workload, student nurses at Wits also had to do the same number of practical hours as their colleagues who trained only at the nursing colleges. These hours were required by the SANC for registration. From the late 19th century until the mid-1980s, nursing education was closely linked to nursing service, and so, these hours of active ward duty were seen as important by the profession. It was, after all, the profession, through the guise of the SANC, that controlled aspects of nurses training even within the universities [26]. This demand for service hours meant that Wits degree students had to perform 40 days of practical training at the Johannesburg General Hospital in their first year. This was all done during their holidays and on Saturday mornings [32]. The link to this professional service was not all bad. It allowed degree students to share some of the benefits of other nursing students. For example, they received a training allowance of about R100 a month, out of which they could pay their university tuition fees and the R272 a year for board and lodging. At this time, many of the Wits degree nurses stayed at the BG Alexander Nurses' Home, where many diploma nurses from BG Alexander collage also lived [23, 32]. 

The 1970s was a decade of consolidation for the nurse educators at Wits. As already mentioned, the Allied Medical Disciplines Committee came into being in 1970. Three years later, the (sub-) subdepartment moved out of the Department of Preventative and Promotive Medicine and became a subdepartment directly under the Dean of the Faculty of Medicine. Williamson, who was then a senior lecturer, was appointed as the temporary head of the sub-department and took up a seat on the University's Senate Committee [7]. It was now only a short step to the establishment of a full and independent Department of Nursing at Wits. This eventually occurred in 1977, and Williamson became Wits' first Professor of Nursing [7, 14]. One of the provisions for granting full departmental status was that all future appointments would be joint appointees of the University and the Transvaal Provincial Administration which was responsible for provincial health policy [7]. While these joint appointments made it possible to employ the faculty that was needed to create a fully fledged Department of Nursing, they were also problematic and led to a rather high staff turnover. This was because of the differentiation in joint appointment and full university employees in terms of salary. Joint position holders were appointed and paid at the level of an Assistant Director in the public service, regardless of if they were appointed as a lecturer, senior lecturer, or at a professorial level. The implementation, by the Gauteng Department of Health, of the Occupational Specific Dispensation for nurses in November of 2007 has lead to the recognition of lecturer specialty qualifications and a significant improvement in salary levels [34]. 

Despite these issues, the department was growing both in terms of course offerings and the number of staff. In 1974, the Diploma in Nursing Administration was added. By the end of the decade, the Department was offering six programmes and two optional courses for registered nurses, the latter being community nursing and psychiatric nursing [7]. By this time, the department was also benefiting by its ability to hire its own graduates. The first appointment of this kind was made in 1978, when Miss RJ Howard was appointed as a tutor. In the years that followed, more former students, such as PA McInerney, NA Abelson, VJ Pinkney, D Lee, and Jill von Dongen, were hired [7].

The 1980s saw both the physical relocation of the Department of Nursing to the Medical School premises in Parktown and major shifts in nursing education. It was in the early years of the 1980s that changes to the professional approach and practical requirements for the student nurses in the Wits' degree programme occurred. The number of practical hours that the SANC demanded from nurses in the Wits degree programme was finally reduced to a more manageable level [7]. These practical hours were still done within the hospital system. Williamson was well aware that the ward sisters did not want students who were studying at university, and even the doctors felt that it was unnecessary for nurses to study at a university [29]. For Williamson, a highlight of her career was when a ward sister said to her: “When are you sending me your girls again?” [29]. At the same time, as changes were being made to the nursing service component of the degree, the SANC who were still in control of nursing education were working on expanding the content of the degree and lengthening the course from three to four years [2]. Williamson remembered that six months after she began at Wits, the SANC called for the university to add a six-week introductory course in psychiatric nursing, midwifery, and public health to the general nursing degree [29]. 

In June 1982, the National Department of Health and Welfare agreed to the integration of nursing education into the university system [35]. This integration became obligatory in 1985 and meant that nursing education was finally separated from the nursing service. This was done though the passing of SANC regulation R425 all nursing colleges became associated with universities. Rather than being subordinated to the hospitals, nursing colleges became autonomous units in 1986. In practice, this meant that colleges were controlled by their own Council and Senate. They had their own budgets, conducted their own academic education (in most cases, a 4-year integrated diploma course leading to nursing registration) and set their own examinations [36]. The colleges were, however, under the academic guardianship of the universities. The Department of Nursing at Wits thus became responsible for the monitoring and controlling of standards at B.G. Alexander Nursing College and the Baragwanath Nursing College, as well as the nursing college at the University of Bophuthatswana in Mafikeng. The Wits Department was also responsible for inspecting the colleges, representing them on the university's Senate, and moderating their examinations. This approach might have shored up academic standards, but it put a great deal of extra pressure on the Wits nursing department [16]. Despite the heavy workload involved in overseeing the colleges and the increased complications of coordinating this new relationship, some seemed to view these changes in a favourable light. 

One of the tangible benefits for the department was the eventual introduction of a number of new lecturer posts. Others saw the interesting new horizons these relationships provided. Robertson described it as “a privilege to have been part of the growth and development of these colleges and to learn with them as they wrestled with new roles, student unrest and changes in composition of student bodies” [37]. Within the nursing administration Charlotte Searle stressed that this meant that not only the nurses who were enrolled in the degree programme, but all nurses, would benefit from the link to the university [26]. At the same time, Marks has shown, with reference to South African nursing more broadly, that the changes led to a further professionalization of nurses' education and the training of a new type of nurse. This “new” nurse was self-sufficient and was comfortable in a primary health care setting as well as in the increasingly technical hospitals [2]. 

Not everyone was supportive of the direction in which nursing education was headed during the 1980s. Many outside of the nursing sphere were still concerned about the shortage of white nurses who were qualifying and did not foresee the changes outlined above as a solution [2]. Others, such as Phillip Tobias from Wits, began to call for a reduction in racial restrictions on nursing posts. He pointed out that: “[T]here is a waiting list of Black nurses seeking jobs at Baragwanath Hospital, while 48% of nursing posts at Johannesburg Hospital are unfilled at present” [15]. The dire shortage of nurses did in fact lead to the relaxing of the criteria for black nursing students to gain admission to Wits and Wits-controlled colleges by the late 1980s.

Finally, there were also staff changes in the 1980s. By 1983, the staff complement had grown to a professor, department head, and six lecturers [7]. In March of 1986, Williamson retired and became Emeritus Professor. Professor Barbara Robertson, who was a rather reluctant addition to the department, took over the helm on 1 April 1986. She had been part of the Department of Nursing at the University of South Africa (UNISA) and was not looking to move when she was headhunted by Wits and invited to come to the university to chat about the post. Robertson recalled arriving for the “chat” and finding about 30 people in the room ready to interview her [29]. Even after the “interview”, Robertson was still reluctant to take the post but Charlotte Searle, who was then head of nursing at UNISA and a rather larger-than-life, powerful figure in the South African nursing arena convinced her to take the post. Searle's justification, as Robertson remembered it, was that Robertson should not let the English-speaking Department down and that if she did not take the post the Department might be closed [29]. This pressure, despite its patronising nature and Robertson's own religious beliefs led her to accept the post at Wits [29].

On her appointment, Robertson was given an ultimatum: to either get the department going or see it closed down [29]. Looking back at the period over a decade later, Robertson put the challenges faced by the department in a succinct and meaningful way which is worth quoting at length.

It was a small department, understaffed, mainly female, and underfinanced. It was a small cog in the large male-orientated medical school, where the training of medical students, specialist physicians and research were paramount issues. Medical personnel found it difficult to accept that nursing was a microcosm of medicine and that within it, there were separate disciplines which could not be taught by the same lecture. This resulted in her being only one lecturer available for each major discipline who in addition to teaching had to supervise the practical learning experiences. High teaching loads mitigated against time for research, and the department had frequently to endure criticism from their medical colleagues [37].

These challenges might not have been unique in the realm of nursing education. However, it seems, at least from Robertson's own recollection, that she was up to the challenge. Within six months, she convinced the Dean to increase the Department's budget and the number of staff by the equivalent of one full-time and one part-time position persons [7]. This was particularly important given the recent restructuring of the relationship between the nursing colleges and the university. 

In the early years of Robertson's headship, the undergraduate degree program was graduating small numbers of students who had completed a degree which placed a heavy emphasis on the sciences [38]. During the later years of the 1990s, Robertson was also instrumental in bringing problem-based learning (PBL), which she had encountered during one of her trips to North America, to the nurses at Wits. Her motivation for introducing PBL to Wits was to see nursing move from rote learning to a more integrated style of learning that addressed topics in a more holistic way. In the years that followed, Robertson arranged for Gayle Langley and Patricia McInerney to undertake an international study tour in order to expand their knowledge of PBL. This eventually became the major teaching and learning approach in the Department [29]. PBL puts much less emphasis on content and focused on encouraging students to take responsibility for their learning. This strategy is still used in the departments' classrooms, but it has been adapted over the years to suit the particular constraints of this type of problem based learning being used alongside a “traditional” lecture based university courses such as biology, pharmacology, anatomy and physiology [39]. 

1994 heralded a new era not only in South Africa's political arena but also in health care. The same year also saw the introduction of a new curriculum for nursing education at Wits. The curriculum was not only based on PBL but also took a more community orientated approach. This shift in programming also corresponded well with the new ANC governments' focus on building up the much neglected Primary Health Care system. The first graduates from this program graduated in 1998 [38].

Professor Barbara Robertson, who had shepherded the department though the exciting but unpredictable years of the birth of the new South Africa, retired in 1997. A governing committee, under the leadership of Dr. Patricia McInerney, was then appointed. More curriculum changes were experienced at this time. The SANC decreased subsidies to the university which in turn lead to a reduction of training from four and a half to four years. All of the changes outlined above were symbolised in the changing of the name of the degree from B.Sc. Nursing to B. Nursing [38].

In July 2001, Professor Hester Klopper was appointed as Head of Department. She describes the three corner stones of the department as education, research and service delivery [38]. Education was of course, central to the entire history of the Department, as was service delivery to some extent. However, the nature of the latter changed over the years. In the 1980s, the department was involved in outreach though its relationship with the University of Bophuthatswana. The outreach and service delivery efforts have been intensified over the last two decades. The department has been involved in a range of service delivery projects both in Gauteng and further afield. These include the Health Services Development Unit (HSDU) at Tintswalo Hospital in the then Eastern Transvaal, the Muldersdrift Clinic, Alexandra Clinic and the Hillbrow Primary Health Care Project. Each week, department staff members spent time in these clinical settings, with the aim of both skill development and service delivery. Work in other clinical settings, such as the Tembisa Hospital Effective Care Research Unit (THECRU) which focuses on women's health, allows staff and students to develop their research programs. Unlike the service component in the hospitals in earlier decades these outreach programs allow for a much greater level of participatory learning and play an important role in delivering services to the most needy.

Research by the staff and graduate students has only more recently come to the forefront of the work of the department. Although postgraduate degrees at both a Masters and Doctoral level were in place by the mid 1970s, the number of students enrolled in these degrees at Wits remained limited. The already overburdened staff did little to develop the program at this stage. When asked about the graduate program during her headship, Robertson remembered that while graduate supervision was seen as important they “couldn't push everything!” [29]. 

From the late 1990s, the M.Sc. in Nursing expanded considerably to include a growing number of specializations. This was followed by a major growth in student intake. Another significant development in the M.Sc. program was the Diploma in Advanced Nursing, developed under the leadership of Patricia McInerney. This development allowed diploma nurses to upgrade their qualifications and to enter the M.Sc. program. This had a profound effect in increasing the levels of access to post-graduate work for nurses from previously disadvantaged backgrounds.

There has also been an expansion of Wits' Ph.D. in Nursing Science. The Ph.D. now comprises of both a course work component, focusing on advanced methodologies, and a research component. There have been more than 10 Ph.D. students [34]. 

Starting with only five students in 1969, the department was filling its quota of 40 students each year by the mid-1990s [40]. By 1990, Wits had graduated about 171 B.Sc. (Nursing) students and these numbers continued to grow [7]. Yet, there continued to be a severe shortage of nurses not only in hospitals but also in educational institutions such as Wits. One of the problems facing the department is the difficulty of filling senior nursing education positions. This is especially acute at universities where lecturers are expected to hold a masters degree. While, as outlined above, increasing numbers of nurses are obtaining masters and doctoral degrees, this has not translated into more candidates wishing to teach at universities [34].

Another recent development was the reintegration of South Africa into international nursing bodies. Some of these international nursing bodies, like the International Network for Doctoral Education in Nursing that was joined in the late 1990s by the Department of Nursing at Wits, had an important bearing on doctoral candidates' global competency. 

The department was internationalising in other ways too. By the new millennium just over 10% of the post-graduate nursing education students were from South African Development Countries (SADC). At the same time, the department has become increasingly involved in nursing education thought out the African continent.

Not only do nursing educators from Wits serve on the executive of the African Honour Society for Nurses (AHSN), they have also used their involvement in the society to develop nursing and midwifery on the continent. For example, members of the department have been involved in mentoring nurse educators at the University of Malawi and have been involved in deploying New Partnerships for African Development (NEPAD) funding to develop nursing and midwifery training in Niger, one of the world's poorest countries [41]. Overseeing these later developments was the fifth Head of Department, Dr. Judith Bruce. The Department of Nursing Education is now part of the School of Therapeutic Science, based in the Faculty of Health Science. What has remained the same is that Wits continues to produce some of the country's most influential nursing leaders. A look back allows one to recognise the efforts of the Wits nursing pioneers and to see the constant changes that have characterized the department. 

This historical overview of the development of nursing education at one of South Africa's major universities serves as a detailed case study of the multiple influences which shaped the development of nursing in South Africa. The Wits case study shows that the push for nursing education to be based within the university system originated from two specific groups. The nurses themselves saw university education as important in the recognition of nursing as a profession. In this way, the Wits nurses fitted in with a broader push by other nurses within South Africa and indeed worldwide. At the same time, the university and medical schools more generally saw the advantages of increasing the training of nurses within the local setting. In the early 20th century, most of the sister tutors were trained abroad. Bringing this training within the ambit of South African universities allowed for the creation of a South African-trained group of nurses who would be able to take nursing in the country into the 20th century in a way that fulfilled local needs. In these ways, nursing education was reinvented to respond to challenges locally and abroad, within the nursing profession and the medical profession more generally. Today, as nursing departments face threats of closure in both developed and developing countries, it is worth looking back at the way departments have met these challenges in the past and what lessons this historical narrative can have for today.
